# A Qualitative Study of Maternal and Caregiver Perceptions of Dietary Practices Contributing to Undernutrition Among Children Under Five in Ngqeleni, Eastern Cape

**DOI:** 10.3390/ijerph23040482

**Published:** 2026-04-11

**Authors:** Patiswa Mto, Xolelwa Ntlongweni

**Affiliations:** 1 School of Public Health, Walter Sisulu University, Mthatha 5117, South Africa; patiswamto@gmail.com; 2 WSU Society and Health Research Institute, Walter Sisulu University, Mthatha 5117, South Africa; 3 WSU Institute for Clinical Governance & Healthcare Administration, East London 5247, South Africa

**Keywords:** caregiver perceptions, child nutrition, maternal and child health, public health nutrition, qualitative research, rural health, South Africa, undernutrition

## Abstract

**Highlights:**

**Public health relevance—How does this work relate to a public health issue?**

- Undernutrition remains a persistent public health problem among children under five in rural communities of the Eastern Cape Province.
- Caregiver perceptions provide critical insights into the socioeconomic and feeding-related determinants of child undernutrition.

**Public health significance—Why is this work of significance to public health?**

- The study highlights how poverty, food insecurity, and limited dietary diversity shape child nutrition outcomes in resource-limited settings.
- Understanding caregiver experiences can inform culturally and contextually appropriate nutrition interventions.

**Public health implications—What are the key implications or messages for practitioners, policy makers and/or researchers in public health?**

- Integrated interventions combining nutrition education with strategies addressing poverty and food access are needed.
- Policies aimed at improving household food security and supporting caregivers may contribute to reducing child undernutrition in rural settings.

**Abstract:**

Background: Undernutrition among children under five years remains a major public health challenge, particularly in low- and middle-income countries and rural communities where poverty, food insecurity, and limited access to health services persist. Maternal and caregiver perceptions play a critical role in shaping feeding practices and health-seeking behaviours that influence child nutritional outcomes. Objective: This study explored mothers’ and caregivers’ perceptions of factors contributing to undernutrition among children under five years in a rural community of Ngqeleni, Eastern Cape, South Africa. Methods: A qualitative descriptive study was conducted at a primary healthcare clinic in the Ngqeleni sub-district. Purposive sampling was used to recruit mothers and caregivers of children under five years. Data were collected through seven in-depth interviews and three focus group discussions involving a total of 25 participants. Interviews were conducted using a semi-structured guide and analyzed thematically. Results: Five major themes emerged: caregivers’ perceptions of nutrition, household food insecurity and unemployment, limited dietary diversity, culturally influenced feeding practices, and gaps in practical nutrition knowledge. Caregivers demonstrated concern for child nutrition but described constrained feeding choices shaped by poverty, reliance on social grants, environmental challenges, and limited access to diverse foods. Environmental challenges such as drought and lack of piped water further limited food production. Limited nutrition knowledge and reliance on informal information sources contributed to suboptimal feeding practices. Conclusions: Undernutrition in this rural setting is shaped by a complex interaction of economic hardship, environmental constraints, and limited caregiver knowledge. Community-based nutrition education, strengthened primary healthcare counselling, and multisectoral interventions addressing poverty, water access, and food security are essential to improve child nutrition outcomes.

## 1. Introduction

Undernutrition among children under five years of age remains one of the most persistent global public health challenges. Despite progress in reducing extreme hunger, malnutrition continues to contribute substantially to childhood morbidity and mortality, particularly in low- and middle-income countries (LMICs) [1,2]. Globally, an estimated 148 million children under five are stunted, 45 million are wasted, and micronutrient deficiencies remain widespread [1]. Undernutrition is associated with nearly half of all deaths among children under five, primarily through increased vulnerability to infectious diseases [2]. Beyond survival, early-life nutritional deprivation impairs cognitive development, educational attainment, and long-term economic productivity, perpetuating intergenerational cycles of poverty and poor health [3].

Sub-Saharan Africa continues to experience a high burden of child undernutrition, with structural poverty, food insecurity, and health system constraints acting as major drivers [1,4]. Although South Africa is classified as an upper-middle-income country, deep socioeconomic inequalities persist, particularly between urban and rural communities. National data indicate that approximately one in four South African children under five are stunted, with a higher prevalence observed in poorer provinces such as the Eastern Cape [5]. Rural households face disproportionate challenges related to unemployment, limited income-generating opportunities, and constrained access to diversified food markets [6]. The Eastern Cape Province is characterized by high levels of poverty and reliance on social grants as the primary household income [6]. Many rural households depend on small informal food retailers, seasonal employment, and subsistence agriculture. These structural constraints influence food purchasing patterns, dietary diversity, and feeding practices within households. Evidence suggests that dietary intake among young children in rural South Africa is often dominated by maize-based staples, with limited consumption of animal-source proteins, fruits, and vegetables, increasing the risk of both macronutrient and micronutrient deficiencies [7].

The UNICEF conceptual framework for malnutrition provides a comprehensive model for understanding these dynamics [4]. The framework distinguishes between immediate determinants (inadequate dietary intake and disease), underlying determinants (household food insecurity, inadequate care and feeding practices, poor health services, and unhealthy environments), and basic determinants (economic structures, political context, and social inequities). In rural South African settings, these determinants interact in complex ways. Chronic poverty constrains access to diverse diets; cultural beliefs and caregiving norms influence feeding behaviors; and health system interventions may not adequately address contextual realities.

Infant and young child feeding (IYCF) practices are central to child nutritional status. The World Health Organization (WHO) recommends exclusive breastfeeding for the first six months of life, followed by the timely introduction of safe, adequate, and nutritionally appropriate complementary foods while continuing breastfeeding up to two years and beyond [8]. However, implementation of these recommendations is often hindered by socioeconomic pressures, perceived breastmilk insufficiency, limited dietary diversity, and inadequate context-specific counseling [9].

Maternal and caregiver perceptions play a critical role in shaping feeding practices. Decisions regarding breastfeeding duration, complementary feeding initiation, portion sizes, and food allocation are embedded within cultural norms, household power dynamics, and economic realities. Studies have shown that knowledge of recommended feeding practices does not necessarily translate into optimal behavior when households face food insecurity and financial instability [10]. Understanding caregivers’ lived experiences is therefore essential for designing effective and contextually appropriate nutrition interventions.

While quantitative surveys in South Africa have documented the prevalence of stunting and food insecurity, fewer studies have explored caregivers’ perspectives on the underlying factors contributing to undernutrition in rural Eastern Cape communities. Qualitative inquiry enables deeper exploration of how structural deprivation, fluctuating income, social grant cycles, and cultural norms influence daily feeding decisions. Such insight is critical to inform multisectoral strategies that address both immediate and structural determinants of child undernutrition.

This study, therefore, aimed to explore mothers’ and caregivers’ perceptions of factors contributing to undernutrition among children under five years in the rural community of Ngqeleni, Eastern Cape. By situating feeding practices within broader socioeconomic and structural contexts, the study contributes to a more nuanced understanding of child undernutrition and provides evidence to inform context-sensitive nutrition policies and interventions.

Unlike previous studies that primarily quantify the prevalence of child undernutrition, this study provides in-depth, context-specific qualitative evidence on how caregivers in a rural Eastern Cape community perceive and negotiate the structural, cultural, and economic constraints shaping child feeding practices, using the UNICEF framework as an interpretive lens.

## 2. Materials and Methods

### 2.1. Study Design

A qualitative approach was selected because it enables exploration of socially embedded feeding behaviors, contextual constraints, and culturally mediated decision-making processes that are not fully captured through quantitative surveys. This study employed an exploratory qualitative design using in-depth individual interviews to examine maternal and caregiver perceptions of dietary practices contributing to undernutrition among children under five years.

**Figure 1:**
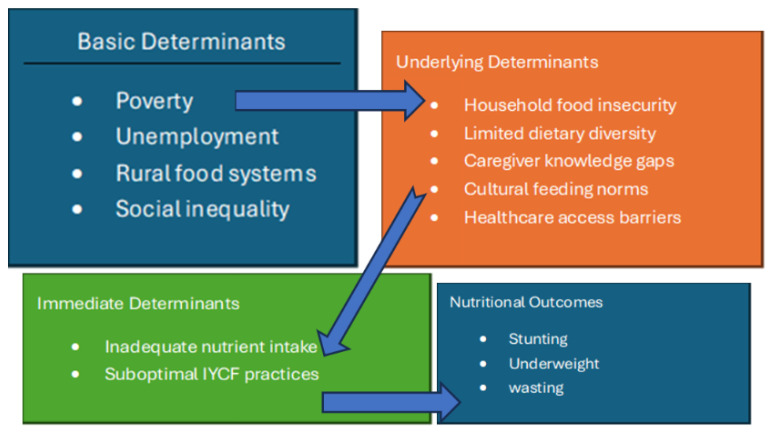
Conceptual framework illustrating the determinants of child undernutrition in rural Ngqeleni, adapted from the UNICEF conceptual framework of malnutrition.

The study was informed by the UNICEF conceptual framework of malnutrition, which recognizes the interaction between basic (structural), underlying (household and community), and immediate (dietary and disease-related) determinants of undernutrition. This framework guided the development of the interview guide and thematic analysis, and the interpretation of findings. Emergent themes were deliberately mapped onto these three levels of determinants, resulting in the conceptual framework presented in Figure 1.

### 2.2. Study Setting

The study was conducted in Ngqeleni, a rural community in the Eastern Cape Province of South Africa. The area is characterized by high unemployment, reliance on subsistence agriculture, and dependence on social grants. Access to diversified food markets is limited, and many households depend on small informal retailers. Primary healthcare services are delivered through local clinics offering maternal and child health services, including growth monitoring and nutrition counseling.

### 2.3. Study Population and Sampling

Participants were mothers and primary caregivers of children under five years residing in Ngqeleni (South Africa). Purposive sampling was used to recruit caregivers attending local primary healthcare clinics for child health services. Recruitment was conducted at the clinic by the researcher with the assistance of clinic staff. Potential participants were identified among caregivers attending child health services and were approached in the waiting area. The study purpose and procedures were explained, and those who expressed interest were screened for eligibility. Written informed consent was obtained prior to participation. Interviews were scheduled at a time convenient for participants, either on the same day or at a later agreed date. Sampling aimed to ensure variation in children’s ages (ranging from infancy to five years) and caregivers’ sociodemographic characteristics (including age, relationship to the child, and socioeconomic background) in order to capture diverse perspectives on child feeding practices and undernutrition.

Recruitment continued until thematic saturation was achieved, defined as the point at which no new themes emerged from additional interviews. Inclusion criteria:

- Aged 18 years and older
- Primary caregiver of a child under five years
- Resident in Ngqeleni for at least six months
- Able to provide informed consent

Exclusion criteria:

- Caregivers who were severely ill or distressed at the time of data collection
- Caregivers unable to participate in an interview due to communication barriers (e.g., severe hearing impairment or language limitations not supported by the research team)
- Caregivers who had previously participated in the study

The focus on primary caregivers ensured that participants had direct responsibility for daily feeding decisions.

### 2.4. Data Collection Procedures

Participants were approached at the clinic (Ngqeleni, Eastern Cape, South Africa) and provided with detailed information about the study, including its purpose, procedures, and their rights as participants. Those who expressed interest were given an opportunity to ask questions, and written informed consent was obtained prior to participation.

A total of 25 participants were included in the study. Data were collected through seven in-depth individual interviews and three focus group discussions (FGDs), each comprising six participants. The FGDs were organized to capture diverse caregiving perspectives: one group included mothers of children aged 0–24 months, the second included mothers of children aged 2–4 years, and the third included other primary caregivers such as grandmothers, guardians, and aunts of children under five years.

Semi-structured interviews were conducted in isiXhosa by trained researchers. An interview guide explored:

- Household food purchasing and access patterns
- Daily meal composition and dietary diversity
- Breastfeeding and complementary feeding practices
- Perceptions of causes of undernutrition
- Cultural beliefs related to food and child growth
- Experiences with nutrition counselling and healthcare services

In-depth semi-structured interviews were conducted with mothers and caregivers, allowing participants to express their views freely while ensuring that key topics related to undernutrition were explored. Focus group discussions complemented individual interviews by capturing shared experiences, community norms, and collective perceptions.

All interviews and FGDs were audio-recorded with participants’ consent and supplemented with field notes. Audio recordings were transcribed verbatim and translated into English where necessary. Transcripts were anonymized by removing identifying information and assigning unique participant codes.

All data were securely stored on password-protected devices accessible only to the research team. Transcripts were reviewed for accuracy and completeness prior to analysis.

Interviews lasted approximately 45–60 min and were audio-recorded with consent. Field notes captured contextual observations.

Data collection took place between June 2025 and August 2025.

### 2.5. Data Analysis

Audio recordings were transcribed verbatim into English. Thematic analysis followed Braun and Clarke’s six-step framework:

- Familiarization with the data
- Initial code generation
- Searching for themes
- Reviewing themes
- Defining and naming themes
- Producing the report

Initial coding was conducted inductively. Codes were then grouped into categories and broader themes reflecting structural, behavioural, and sociocultural determinants of undernutrition. Themes were developed iteratively and organized; the UNICEF conceptual framework of malnutrition informed the analytical process by guiding the organization of codes and the mapping of emergent themes into structural, underlying, and immediate determinants. This process informed the development of the conceptual framework presented in Figure 1. To enhance trustworthiness, peer debriefing and independent code verification were performed. Each participant was assigned a unique identification code to maintain anonymity. All electronic files were password-protected and stored on a secure device accessible only to the research team.

### 2.6. Trustworthiness and Rigor

To enhance credibility, dependability, confirmability, and transferability, the following strategies were implemented:

- Triangulation: Use of interview data and field notes
- Peer debriefing: Regular supervisory discussions during analysis
- Audit trail: Documentation of coding decisions and theme development
- Reflexivity: The researcher maintained a reflexive journal to acknowledge positionality and potential biases
- Member reflection (if applicable): Selected participants were asked to verify interpretations

These measures strengthened analytic rigor and minimized subjective bias.

The researcher’s positionality was explicitly considered throughout the study. Data collection was conducted by the first author, a professional nurse with training in public health and experience in maternal and child health services, who is also a resident of the study community. Shared linguistic, cultural, and social familiarity facilitated rapport and trust during interviews conducted in isiXhosa. At the same time, the researcher’s dual role as both a community member and a health professional may have influenced participants’ responses or encouraged socially desirable answers. To address this, efforts were made to minimize power imbalances by emphasizing the researcher’s role as a learner rather than an authority figure. A reflexive journal was maintained throughout data collection and analysis, and regular supervisory debriefing was undertaken to critically examine how professional training, community proximity, and assumptions about “ideal” nutrition and feeding practices may have shaped data collection and interpretation. 

### 2.7. Ethical Considerations

The study obtained ethical approval from the Walter Sisulu University Human Research Ethics Committee (Reg Number: WSU HREC 056/2025), Nyandeni sub-district office, and Mqanyeni Clinic, Eastern Cape Department of Health Research Committee (EC_202506_020). Written informed consent was obtained from all participants. Confidentiality and anonymity were maintained throughout the study.

Participants were informed that:

- Participation was voluntary
- They could withdraw at any time without consequences
- No identifying information would appear in publications

No financial incentives were provided. Participants requiring additional nutrition support were referred to clinic services where appropriate.

Additional materials are provided in the Supplementary Materials.

## 3. Results

A total of 25 participants were included in the study. Table 1 presents a summary of participants’ demographic characteristics, including age, employment status, and relationship to the child. The majority of participants were mothers and were unemployed, relying primarily on social grants and informal sources of income. Only a few participants reported formal or self-employment, while some households depended on extended family support. Participant characteristics are summarized in Table 2.

### 3.1. Household Food Insecurity and Economic Constraints

Household economic hardship emerged as the dominant structural determinant of undernutrition. Participants consistently described insufficient income, dependence on social grants, and recurring food shortages, particularly toward the end of the month.

Many caregivers reported that food purchased at the beginning of the month did not last: “The grant comes, and we try to buy groceries, but before the month ends, the food is finished. Then we just eat what is left, sometimes only porridge.” (Participant 4, 32 years).

Some participants described periods of complete food scarcity: “There are days when there is nothing in the house. The children ask for food, but there is nothing to give.” (Participant 11, 29 years).

Financial instability directly influenced meal frequency and portion size. Care-givers reported reducing meal portions or skipping meals so that children could eat:

“Sometimes I eat less so that my child can have more. But even that is not enough.”

(Participant 7, 35 years)

These narratives demonstrate that food insecurity is not episodic but chronic, shaping daily feeding decisions and limiting the ability to provide nutritionally adequate diets.

### 3.2. Limited Dietary Diversity and Monotonous Diets

Dietary patterns were characterized by heavy reliance on maize meal and other carbohydrate-dense staples, with limited inclusion of animal-source proteins, fruits, and vegetables. Participants described typical meals as repetitive: “Most days it is pap [maize porridge] and maybe cabbage. Meat is not something we can buy often.” (Participant 2, 27 years). Protein-rich foods such as eggs, chicken, or beans were often consumed irregularly due to cost: “Eggs are expensive now. We buy them only when there is extra money.” (Participant 9, 30 years).

Several caregivers acknowledged awareness that children require a variety of foods but felt constrained: “They tell us at the clinic that children must eat vegetables and fruit, but where will we get money for that?” (Participant 14, 24 years).

The lack of dietary diversity suggests potential micronutrient inadequacies and highlights the tension between knowledge and affordability.

### 3.3. Suboptimal Infant and Young Child Feeding Practices

Breastfeeding initiation was common; however, exclusive breastfeeding for six months was inconsistently practiced. Some mothers introduced complementary foods early due to perceptions of insufficient breast milk. “I started giving soft porridge at three months because I felt my milk was not enough. The baby was crying all the time”. (Participant 6, 22 years).

Complementary feeding practices were often limited in diversity and frequency. Meals for young children frequently mirrored adult diets: “The child eats what we eat. There is no special food for children.” (Participant 1, 31 years).

Feeding frequency was sometimes inconsistent, particularly during periods of food scarcity: “If there is little food, we give small portions and maybe only twice that day.” (Participant 10, 34 years).

These practices reflect both resource limitations and gaps in practical feeding guidance.

### 3.4. Nutrition Knowledge and Health Education Gaps

Although most caregivers reported receiving some nutrition information at primary healthcare clinics, many described the guidance as brief or general rather than tailored to their lived circumstances. “They just say give a balanced diet, but they do not explain how when you do not have money.” (Participant 8, 26 years).

Several participants expressed uncertainty about portion sizes and appropriate meal composition: “I do not know how much is enough for a small child. I just give what we have.” (Participant 12, 28 years).

This suggests that existing nutrition education may not sufficiently address practical barriers or provide culturally relevant solutions.

### 3.5. Cultural Beliefs and Household Food Allocation

Traditional beliefs and norms influenced feeding practices. Some caregivers avoided certain foods for young children due to cultural perceptions. “We do not give some foods to small children because elders say it is not good for them.” (Participant 5, 40 years).

In addition, intra-household food allocation sometimes prioritized adults engaged in physically demanding labor: “If there is meat, it is given first to the father because he works. The children eat what remains.” (Participant 13, 36 years).

These dynamics illustrate how social hierarchies within households may affect child dietary intake.

### 3.6. Healthcare Access and Structural Barriers

Although healthcare facilities were geographically accessible to many participants, indirect costs and long waiting times discouraged frequent visits. “Going to the clinic means paying for transport. If there is no money, you stay at home.” (Participant 15, 33 years).

Some caregivers felt that nutrition counseling was not consistently emphasized during clinic visits: “Sometimes they just weigh the child and give medicine. They do not always talk about food.” (Participant 3, 25 years).

These findings indicate missed opportunities for reinforcing preventive nutrition messaging.

### 3.7. Interpretation of Results

Across themes, undernutrition was conceptualized not as a singular problem but as the cumulative outcome of poverty, limited food access, constrained dietary diversity, feeding practices shaped by both necessity and tradition, and systemic service limitations. Caregivers demonstrated concern for their children’s well-being but operated within restrictive socioeconomic environments that constrained dietary choices.

## 4. Discussion

The findings of this study indicate that caregiver knowledge of optimal child feeding practices does not operate in isolation but is mediated by structural and environmental constraints. Rather than reflecting a simple knowledge–practice gap, caregivers’ narratives reveal active decision-making and agency within highly constrained contexts. Caregivers demonstrated a clear desire to feed their children appropriately and drew on both biomedical advice and traditional knowledge; however, poverty, food insecurity, unemployment, and environmental challenges such as drought severely limited the choices available to them. As a result, feeding practices reflected negotiated trade-offs between nutritional ideals and material realities, including prioritization of food quantity over dietary quality and intra-household coping strategies during periods of scarcity. The findings align with the UNICEF conceptual framework of malnutrition, which distinguishes between immediate causes (inadequate dietary intake and disease), underlying causes (household food insecurity, inadequate care practices, limited health services), and basic structural determinants (poverty, inequality, and sociopolitical context) [1]. As illustrated in Figure 1, caregivers’ perceptions revealed interacting basic determinants. The framework highlights how broader socioeconomic conditions shape household food access and caregiving behaviours, which in turn directly influence children’s dietary intake and nutritional outcomes.

### 4.1. Household Food Insecurity and Economic Constraints

Household food insecurity emerged as the dominant structural determinant shaping child undernutrition in this rural context. Caregivers consistently described chronic income insufficiency, dependence on social grants, and recurring food shortages, particularly toward the end of the month. These findings corroborate national data indicating that rural households in the Eastern Cape experience disproportionately high levels of poverty, unemployment, and food insecurity compared to those in urban areas [5,6].

Although social grants play a critical role in alleviating extreme hunger, they are often insufficient to ensure nutritionally adequate and diverse diets for young children [6]. Caregivers’ accounts illustrated how limited income constrained meal frequency, portion size, and food quality, reinforcing evidence that economic deprivation directly undermines child feeding practices in low-resource settings [2,3]. Within the UNICEF framework, household food insecurity represents a key underlying determinant linking broader structural poverty to immediate dietary inadequacy and growth faltering [1,4].

### 4.2. Limited Dietary Diversity and Monotonous Diets

Children’s diets were characterized by a heavy reliance on maize-based staples, with infrequent consumption of animal-source foods, fruits, and vegetables. Similar dietary patterns have been documented in rural South African communities, where limited purchasing power and poor access to diversified food markets constrain dietary choices [7]. Although caregivers demonstrated awareness of the importance of food variety, affordability and availability shaped actual feeding practices.

Low dietary diversity is a validated proxy indicator of micronutrient adequacy among children aged 6–23 months and is strongly associated with linear growth and immune function [8]. Diets dominated by starch-based staples increase the risk of deficiencies in iron, zinc, and vitamin A, which are known contributors to anemia, stunting, and vulnerability to infection [2,3]. These findings highlight the discrepancy between recommended dietary guidelines and the lived realities of caregivers in food-insecure rural environments.

### 4.3. Suboptimal Infant and Young Child Feeding Practices

While breastfeeding initiation was common, exclusive breastfeeding for the first six months was inconsistently practiced. Early introduction of complementary foods was frequently attributed to perceived breast milk insufficiency and infant crying. Similar findings have been reported in other low- and middle-income countries, where maternal perceptions and economic pressures influence deviations from recommended infant and young child feeding (IYCF) practices [9,10].

Complementary feeding practices were often limited in diversity and frequency, with young children consuming the same foods as adults. Such practices fall short of WHO IYCF guidelines, which emphasize the timely introduction of nutritionally adequate complementary foods while continuing breastfeeding [8,11]. Within the UNICEF framework, these feeding practices constitute immediate determinants of undernutrition, closely linked to underlying socioeconomic constraints rather than caregiver neglect or lack of concern [1].

### 4.4. Nutrition Knowledge and Health Education Gaps

Most caregivers reported receiving some nutrition information at primary healthcare facilities; however, the content was often described as general and insufficiently tailored to their socioeconomic circumstances. This reflects the well-documented knowledge–practice gap, where awareness of optimal feeding practices does not translate into improved behaviour in the absence of economic and structural support [12]. Previous studies in similar contexts have shown that nutrition counselling is more effective when it incorporates practical guidance using locally available and affordable foods [10]. The findings suggest that clinic-based nutrition education in this setting may benefit from greater contextualization, including realistic portion guidance, culturally appropriate food examples, and problem-solving strategies for food-insecure households.

### 4.5. Cultural Beliefs and Intra-Household Food Allocation

Cultural beliefs and household norms influenced child feeding practices, including food avoidance based on traditional advice and preferential allocation of nutrient-dense foods to adults engaged in physically demanding labour. Similar patterns have been observed in other sub-Saharan African settings, where social hierarchies and cultural perceptions shape complementary feeding and intra-household food distribution [13,14].

Such dynamics may unintentionally disadvantage young children during periods of scarcity, reinforcing inequalities in dietary intake. Addressing these issues requires culturally sensitive interventions that engage caregivers, elders, and other household decision-makers to promote equitable food allocation and child-centered feeding practices.

### 4.6. Health System Barriers and Missed Opportunities

Although primary healthcare services were geographically accessible, indirect costs such as transport expenses and long waiting times reduced consistent clinic attendance. Caregivers also reported that nutrition counselling was not consistently emphasized during clinic visits. Similar barriers have been identified in rural South African healthcare settings, where structural constraints limit the effectiveness of preventive nutrition services [15]. Integrating routine dietary screening, strengthening growth monitoring follow-up, and expanding community health worker outreach may improve early identification of growth faltering and caregiver support. Health system interventions must be responsive to the socioeconomic realities faced by caregivers to ensure meaningful and sustained engagement.

### 4.7. Implications for Nutrition Policy and Practice

The findings demonstrate that child undernutrition in rural Ngqeleni is the result of interconnected structural, underlying, and immediate determinants. Interventions focused solely on caregiver education or clinic-based counselling are unlikely to achieve sustained impact without complementary strategies addressing poverty, food access, and dietary quality [1,4]. Policy responses should strengthen nutrition-sensitive social protection mechanisms to improve household food sufficiency while explicitly promoting dietary quality for young children. Primary healthcare-based nutrition counselling should be tailored to caregivers’ lived realities by incorporating practical guidance using locally available and affordable foods.

Multisectoral collaboration between health, agriculture, water, and social development sectors is essential to address environmental constraints such as water access and to support sustainable household food production in rural communities.

Viewing the findings through the UNICEF framework highlights the need for integrated interventions that simultaneously address structural poverty, household food environments, and caregiving practices, rather than isolated nutrition education strategies.

Figure 1 illustrates the interaction between basic (structural), underlying (household and community), and immediate (dietary and caregiving) determinants of child undernutrition, as informed by the UNICEF conceptual framework. The framework was used to guide data collection, thematic analysis, and interpretation of findings in this study.

### 4.8. Strengths and Limitations

A key strength of this study lies in its qualitative design, which enabled in-depth exploration of mothers’ and caregivers’ perceptions of dietary practices contributing to child undernutrition within a specific rural context. The inclusion of both mothers and other primary caregivers, as well as the use of in-depth interviews and focus group discussions, enhanced the richness and credibility of the data and allowed for diverse caregiving perspectives to be captured.

This study also has some limitations. It was conducted in a single rural community, which may limit the transferability of the findings to other settings. In addition, participants were recruited through a primary healthcare clinic, potentially excluding caregivers who do not routinely access health services. As with all qualitative studies, the findings are based on self-reported experiences and may be subject to recall or social desirability bias. These limitations were mitigated through rigorous qualitative procedures, including the use of trained interviewers, local language interviews, and strategies to enhance trustworthiness.

## 5. Conclusions

This study provides in-depth qualitative insight into mothers’ and caregivers’ perceptions of dietary practices contributing to undernutrition among children under five years in a rural Eastern Cape community. The findings demonstrate that undernutrition in this setting is not the result of isolated feeding behaviours, but rather the cumulative effect of structural poverty, household food insecurity, limited dietary diversity, culturally influenced feeding practices, and health system constraints.

By applying the UNICEF conceptual framework of malnutrition, the study highlights how basic, underlying, and immediate determinants interact to shape child nutritional outcomes in resource-limited rural contexts. Caregivers displayed concern for their children’s well-being; however, their ability to implement recommended feeding practices was constrained by socioeconomic and environmental realities beyond their control.

These findings highlight the importance of moving beyond narrowly focused nutrition education interventions toward integrated, multi-sectoral approaches that address poverty, food access, dietary quality, water security, and context-appropriate nutrition counselling. Strengthening social protection mechanisms, enhancing community-based nutrition support, and tailoring primary healthcare counselling to caregivers’ lived circumstances may contribute to sustainable improvements in child nutrition outcomes.

This study contributes to contextually grounded evidence that can inform rural nutrition policies and programmes aimed at reducing child undernutrition in South Africa and similar low-resource settings.

## Figures and Tables

**Table 1:** Thematic analysis revealed 5 major themes.

	Major Theme	Sub-Theme
1.	Perception of nutrition	Understanding of nutrition and undernutrition
		feeding beliefs
2.	Socio-economic factors	Financial constraints
		Employment status
		Household support
3.	Cultural beliefs and practices	Traditional beliefs
		Religious dietary restrictions
		Intergenerational advice
4.	Environmental factors	Water access
		Drought
		Subsistence farming
5.	Nutritional knowledge and feeding practices	Sources of information
		Food selection
		Meal frequency
		Perceptions of healthy food

**Table 2:** Summary of Participants’ Characteristics (N = 25).

Participant	Age (Years)	Employment Status	Relationship to Child	No. of Children	Main Source of Income
P1	30	Unemployed	Mother	1	Security guard income + grants
P2	27	Unemployed	Mother	2	Grants + security guard income
P3	38	Informal (piece jobs)	Grandmother caregiver	1	Grants + piece jobs
P4	18	Unemployed	Mother	1	Grant + spaza shop income
P5	Not stated	Unemployed	Mother	2	Husband (cattle herder) + grant
P6	27	Unemployed	Mother	1	Grants
P7	Not stated	Unemployed	Mother	4+	Security income + grants
P8	Not stated	Unemployed	Mother	1	Husband (piece jobs) + grant
P9	21	Unemployed	Mother	1	Pension + grant
P10	43	Unemployed	Mother	4	Husband (bricklayer) + grant
P11	Not stated	Informal trader	Mother	4	Small business + grants
P12	36	Informal (piece jobs)	Mother	2	Piece jobs + grants
P13	30	Unemployed	Mother	3	Husband (intern) + grants
P14	Not stated	Informal (piece jobs)	Mother	4	Piece jobs + grants
P15	26	Employed (family supported)	Mother	1	Family income + grant
P16	40	Self-employed (consultation)	Mother	4	Grants + consultation fees
P17	43	Unemployed	Mother	3	Grants + family support
P18	34	Unemployed	Mother	3	Grants + family support
P19	33	Unemployed	Mother	2	Sister’s salary + foster grant
P20	23	Informal trader	Mother	2	Grants + small business
P21	28	Unemployed	Mother	3	Family support + grants
P22	34	Unemployed	Mother	4	Grants
P23	34	Unemployed	Mother	3	Husband (mine worker) + grant
P24	39	Self-employed	Mother	2	Business + husband’s income
P25	40	Unemployed	Mother	4	Grants + family income

## Data Availability

The de-identified datasets generated and analyzed during this study are available from the corresponding author upon reasonable request. Data are securely stored on password-protected institutional servers and managed in accordance with ethical approvals and participant confidentiality requirements.

## Supplementary Materials

The following supporting information can be downloaded at: https://doi.org/10.5281/zenodo.18748869.

## Abbreviations

The following abbreviations are used in this manuscript:

DHS	Demographic and Health Survey
DOH	Department of Health
IYCF	Infant and Young Child Feeding
LMICs	Low- and Middle-Income Countries
NDoH	National Department of Health
PHC	Primary Healthcare
SDG	Sustainable Development Goal
Stats SA	Statistics South Africa
UNICE	United Nations Children’s Fund
WHO	World Health Organization

## References

[1] UNICEF, World Health Organization and World Bank Group (2023). Levels and Trends in Child Malnutrition: UNICEF/WHO/World Bank Group Joint Child Malnutrition Estimates: Key Findings of the 2023 Edition.

[2] Black R.E., Victora C.G., Walker S.P., Bhutta Z.A., Christian P., de Onis M., Ezzati M., Grantham-McGregor S., Katz J., Martorell R. (2013). Maternal and child undernutrition and overweight in low- and middle-income countries. Lancet.

[3] Victora C.G., Adair L., Fall C., Hallal P.C., Martorell R., Richter L., Sachdev H.S. (2008). Maternal and child undernutrition: Consequences for adult health and human capital. Lancet.

[4] UNICEF (2013). Improving Child Nutrition: The Achievable Imperative for Global Progress.

[5] National Department of Health (South Africa), Statistics South Africa, South African Medical Research Council, ICF (2017). South Africa Demographic and Health Survey 2016: Key Indicators Report.

[6] Statistics South Africa (2023). General Household Survey 2022.

[7] Faber M., Wenhold F., Laurie S. (2013). Dietary diversity and vegetable and fruit consumption of children in a resource-poor setting in South Africa. Int. J. Food Sci. Nutr..

[8] World Health Organization (2009). Infant and Young Child Feeding: Model Chapter for Textbooks for Medical Students and Allied Health Professionals.

[9] World Health Organization, UNICEF (2003). Guiding Principles for Complementary Feeding of the Breastfed Child.

[10] Na M., Aguayo V.M., Arimond M., Stewart C.P. (2018). Risk factors of poor complementary feeding practices in South Asia: A multilevel analysis. Matern. Child Nutr..

[11] Ruel M.T., Alderman H., Maternal and Child Nutrition Study Group (2013). Nutrition-sensitive interventions and programmes: How can they help to accelerate progress in improving maternal and child nutrition?. Lancet.

[12] UNICEF (2019). The State of the World’s Children 2019: Children, Food and Nutrition.

[13] Aubel J. (2012). The role and influence of grandmothers on child nutrition: Culturally designated advisors and caregivers. Matern. Child Nutr..

[14] Pelto G.H., Armar-Klemesu M. (2011). Identifying interventions to help transition complementary feeding recommendations into practice. Matern. Child Nutr..

[15] Schneider H., Besada D., Sanders D., Daviaud E. (2018). Progress in increasing access to primary healthcare in South Africa. S. Afr. Health Rev..

